# A Comprehensive Visual Detection Strategy: Versatile LAMP Assay with Phenol Red and Lateral Flow Dipstick for On-Site Detection of *Riemerella anatipestifer*

**DOI:** 10.3390/microorganisms14051037

**Published:** 2026-05-02

**Authors:** Jiafeng Wu, Nansong Jiang, Qizhang Liang, Hongmei Chen, Rongchang Liu, Qiuling Fu, Guanghua Fu, Chunhe Wan, Ping Wei, Longfei Cheng, Yu Huang, Tianchao Wei, Weiwei Wang

**Affiliations:** 1 Institute for Poultry Science and Health, College of Animal Science and Technology, Guangxi University, Nanning 530004, China; jiafengwukk@163.com (J.W.); weiping@gxu.edu.cn (P.W.); 2 Fujian Key Laboratory for Control and Prevention of Avian Diseases, Fujian Industry Technology Innovation Research Academy of Livestock and Poultry Diseases Prevention and Control, Institute of Animal Husbandry and Veterinary Medicine, Fujian Academy of Agricultural Sciences (FAAS), Fuzhou 350013, China; nansongjiang@126.com (N.J.); qzl1181@zuaa.zju.edu.cn (Q.L.); chenhongmei2025@126.com (H.C.); liurongc@foxmail.com (R.L.); qiulingfu0822@163.com (Q.F.); fuyuan163@163.com (G.F.); chunhewan@126.com (C.W.); lf396@139.com (L.C.); huangyu_815@163.com (Y.H.)

**Keywords:** *Riemerella anatipestifer* (RA), loop-mediated isothermal amplification (LAMP), visual detection, lateral flow dipstick (LFD), phenol red

## Abstract

*Riemerella anatipestifer* (RA) is the primary causative agent of infectious serositis in ducks, causing significant economic losses. In this study, a rapid and visual loop-mediated isothermal amplification (LAMP) assay targeting the conserved region of the *ompA* gene was developed. Specific primers and a FAM-labeled probe were designed, and amplification products were visualized using phenol red-based colorimetric detection and a lateral flow dipstick (LFD) system. Among the five candidate primer sets, primer set 2 was selected because it showed the highest amplification efficiency and specificity, with no cross-reactivity detected against 12 common waterfowl pathogens. Under optimal conditions, the phenol red-based LAMP assay yielded visible results after incubation at 65 °C for 30 min, while the LAMP-LFD assay required an additional 3~5 min probe hybridization step, with detection limits of 7.76 × 10^2^ copies/μL for the phenol red-based method and 7.76 × 10^0^ copies/μL for the LAMP-LFD method. Thirty clinical samples suspected of RA infection were analyzed using conventional PCR and the developed visual LAMP assays. The positive detection rates obtained with the LAMP-LFD and phenol red-based LAMP methods were 63.3% and 60%, respectively, showing high concordance with conventional PCR (56.7%). In conclusion, the LAMP assay integrating phenol red visualization and lateral flow dipstick detection is rapid, sensitive, and easy to perform, and both detection formats show potential for point-of-care or on-site applications, and can be used for the early diagnosis and detection of RA.

## 1. Introduction

*Riemerella anatipestifer* (RA) is the primary causative agent of infectious serositis, with at least 21 serotypes, which can infect a wide range of domestic poultry and wild birds, including ducks, geese, and turkeys. The infectious serositis caused by RA is widely distributed worldwide and represents one of the most severe bacterial diseases affecting the waterfowl industry [1,2]. In meat ducks, RA infection primarily results in characteristic pathological lesions such as pericarditis, perihepatitis, and airsacculitis. Meanwhile in chickens, RA infection frequently leads to caseous obstruction of the oviduct, consequently causing a marked reduction in egg production [3,4]. To date, RA infections have been reported in numerous countries, including China, India, Thailand, Poland, Germany, and Hungary [5,6,7,8,9,10]. Therefore, the development of a simple, rapid, and convenient method for the on-site visual detection of RA is of great importance for early diagnosis, effective disease control, and a reduction in economic losses.

Due to the large number of serotypes of RA, as well as the existence of subtypes within certain serotypes, accurate diagnosis and identification of RA are critical for effective disease control. Currently, the main methods used for RA detection include pathogenic methods, serological assays, and molecular diagnostic techniques. Pathogenic detection primarily involves bacterial isolation and identification, combined with microscopic examination following Gram staining or Wright-Giemsa staining [11]. Serological methods include agar gel immunodiffusion (AGID), agglutination tests, fluorescent antibody tests (FAT), enzyme-linked immunosorbent assays (ELISA), and colloidal gold immunochromatographic assays (GICA) [12,13,14]. Molecular detection methods mainly comprise polymerase chain reaction (PCR), real-time quantitative PCR (qPCR), multiplex PCR (mPCR), and loop-mediated isothermal amplification (LAMP) [15]. However, bacterial isolation is time-consuming and labor-intensive, making it impractical for on-site rapid diagnosis. Serological assays may yield false-positive results due to cross-reactivity caused by shared antigenic epitopes among different pathogens. Although conventional PCR-based methods offer high specificity and sensitivity, their application is limited by the requirement for expensive equipment and skilled personnel. Therefore, the development of a rapid and accurate on-site visual diagnostic method is essential for the effective control of RA infections [16].

LAMP technology was first established by Notomi et al. in 2000 and needs two pairs of specific inner and outer primers targeting the gene of interest, enabling rapid and efficient nucleic acid amplification through the strand displacement activity of *Bst* DNA polymerase [17]. Because the reaction proceeds under isothermal conditions (60~65 °C), it can be performed using simple equipment such as a water bath or heating block. In 2011, Zheng et al. developed a LAMP assay for the detection of RA; however, the amplification products were typically analyzed by agarose gel electrophoresis or turbidity measurement, which are associated with an increased risk of false-positive results, high instrument costs, and limited applicability in primary-level settings [18].

**Figure 1:**
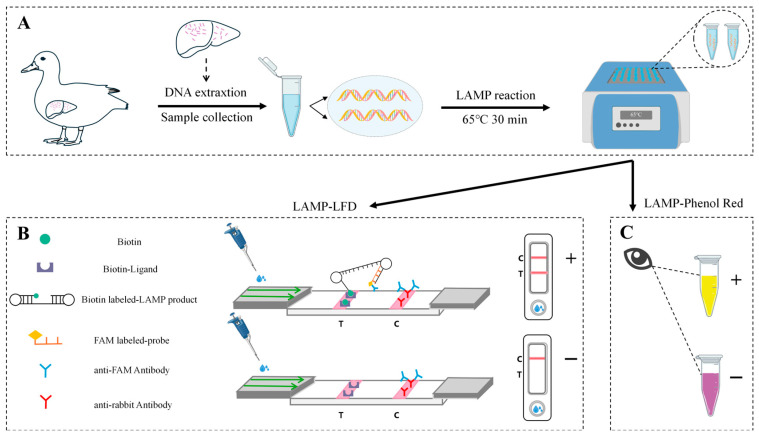
**Workflow of LAMP assay with phenol red and LFD for on-site Detection of RA.** (**A**) Schematic diagram of sample collection, DNA extraction and amplification. (**B**) LFD-based LAMP assay. (**C**) Phenol red-based LAMP assay.

In this study, a LAMP-based assay was developed by integrating two parallel and complementary detection strategies: lateral flow dipstick (LFD) analysis and phenol red-based colorimetric visualization (Figure 1). The first strategy relies on the specific hybridization between a FAM (carboxyfluorescein)-labeled probe and biotin-labeled LAMP amplicons, allowing visual detection and result interpretation through immunochromatographic reactions on the LFD. The second strategy employs phenol red as a pH indicator, which undergoes a color transition from magenta to orange yellow as the pH of the reaction mixture decreases, thereby enabling real-time visual detection of target nucleic acid amplification without the need to open the reaction tube. The combined application of these two methods retains the high specificity of LFD detection while enhancing convenience and real-time monitoring through phenol red-based visualization, together establishing an intuitive and reliable dual-verification detection system. Moreover, this method does not require toxic reagents such as ethidium bromide or complex instrumentation, and demonstrates rapid detection, high specificity and sensitivity, good reproducibility, and equipment independence. As such, it provides a novel approach for the rapid and early detection of RA and for large-scale, real-time epidemiological surveillance.

## 2. Materials and Methods

### 2.1. RA Strains, Clinical Samples and DNA Extraction

All the RA strains used in this study, including RA816, were isolated and purified with TSA agar medium from tissues and organs of ducks or chickens suspected of RA infection and were stored in lyophilized form at −20 °C. A total of 30 clinical samples were collected between 2023 and 2025 from Guangxi, Fujian, Henan, and Shandong Provinces, China. These samples were obtained from ducks or chickens showing clinical signs such as pericarditis, perihepatitis, airsacculitis, and caseous obstruction of the oviduct. Genomic DNA from all strains was extracted using a bacterial genomic DNA rapid extraction kit (Sangon Biotech, Shanghai, China) and stored at −20 °C for subsequent use.

### 2.2. Primer and Probe Design

To ensure assay specificity, primers and probes were designed based on the conserved region of the *ompA* gene of *RA* retrieved from GenBank (accession no. CP121210.1). Five sets of LAMP primers were designed using the NEB online LAMP primer design tool (New England Biolabs, Ipswich, MA, USA) in accordance with standard LAMP primer design principles. These candidate primer sets were subsequently evaluated by PCR, and the set showing the highest specificity and amplification efficiency was selected for further analysis. For lateral flow dipstick (LFD)-based detection, the 5′ end of the forward inner primer (FIP) of the optimal primer set was labeled with biotin (BIO). In addition, a FAM-labeled probe was designed within the target region of the selected primer set to specifically hybridization with the LAMP amplification products. All primers and probes are listed in Table 1 and were synthesized by Sangon Biotech (Sangon Biotech, Shanghai, China).

### 2.3. Construction of Recombinant Plasmid

PCR primers targeting the *ompA* gene were designed, and amplification was performed using RA DNA samples as templates. The PCR products were analyzed by 2% agarose gel electrophoresis, purified using a DNA gel extraction kit (Magen Biotechnology, Guangzhou, China), and subsequently cloned into the pMD18-T vector to construct recombinant plasmids. The resulting construction was confirmed by PCR and DNA sequencing. Plasmid DNA was then extracted using an endotoxin-free plasmid miniprep kit from (Solarbio, Beijing, China), and the concentration and purity were determined using a BioDrop microvolume spectrophotometer. The recombinant plasmid was designated as pMD18T-RA. The plasmid copy number was calculated using the following formula: plasmid copies/μL $=\frac{6.02\times 10^{23}\times (C\times 10^{-9})}{L\times 660}$. C represents the concentration of the recombinant plasmid.

### 2.4. Establishment of the LAMP Reaction System and Primer Set Screening

The LAMP assay was established in a total reaction volume of 25 μL according to the manufacturer’s instructions (EZassay Biotech, Shenzhen, China). To minimize the risk of contamination, all reaction mixtures were prepared using aerosol-resistant filter tips (Bioland, Hangzhou, China). Each reaction contained 12.5 μL of 2× Colorimetric LAMP Reaction Buffer, 1 μL of 25× Colorimetric LAMP Enzyme Mix, 2.5 μL of 10× primer mixture (final concentrations: 2 μM each of F3/B3, 16 μM each of FIP/BIP, and 4 μM each of LF/LB), 1 μL of DNA template, and DNase-free ddH_2_O to a final volume of 25.0 μL. The amplification was performed at 64 °C for 30 min, followed by enzyme inactivation at 80 °C for 5 min to terminate the reaction. The amplified products were then analyzed by agarose gel electrophoresis. The primer set that generated the clearest and brightest characteristic ladder-like bands was selected as the optimal and was used for subsequent optimization of reaction conditions.

### 2.5. Optimization Procedure for LAMP Reaction Conditions

The reaction conditions of the LAMP assay were optimized using a single-factor experimental design. Specifically, the ratio of inner to outer primers (ranging from 1:1 to 16:1), incubation time (10~60 min), and reaction temperature (61~66 °C) were evaluated sequentially to identify the optimal amplification parameters. In each experiment, DNase-free ddH_2_O was used instead of template DNA as the negative control. The amplification products were evaluated by 2% agarose gel electrophoresis and phenol red-based colorimetric visualization. The optimal reaction conditions were determined based on the amplification efficiency and clarity of the visual readout.

### 2.6. Lateral Flow Dipstick (LFD) Detection

For LFD-based detection, LAMP amplification was performed using a biotin-labeled forward inner primer (FIP). After amplification, the reaction was not terminated by heat inactivation. Instead, 20 pmol of the FAM-labeled probe RA2-HP was added directly to the reaction mixture, followed by probe hybridization at 65 °C for 5 min. Subsequently, 5 μL of the hybridization product was mixed with 80 μL of assay buffer (Milenia Biotec GmbH, Giessen, Germany). A lateral flow dipstick (Milenia Biotec GmbH, Giessen, Germany) was then inserted into the mixture and allowed to develop for 3~5 min. The test results were interpreted visually. The appearance of both the control line (C) and the test line (T) as red bands was considered a positive result. The presence of only the control line (C) indicated a negative result, whereas the absence of the control line indicated an invalid result.

### 2.7. Phenol Red-Based Visual Detection of LAMP Detection

The Colorimetric LAMP Reaction Buffer (2×) contained phenol red as a pH-sensitive indicator, enabling direct visual interpretation of amplification results. LAMP reactions were performed under the optimized conditions described above. After amplification, a magenta-colored reaction mixture was interpreted as a negative result, whereas a color change to orange yellow indicated a positive result.

### 2.8. Sensitivity Analysis of Conventional PCR and LAMP-LFD Detection

The sensitivity of the LAMP-LFD assay was evaluated and compared with that of conventional PCR. The recombinant plasmid pMD18T-RA was serially diluted 10-fold, ranging from 7.76 × 10^7^ copies/μL to 7.76 × 10^−4^ copies/μL, and the resulting dilutions were used as templates for both LAMP-LFD and conventional PCR amplification. The conventional PCR assay was performed in a total volume of 25 μL containing 12.5 μL of 2× Rapid Taq Master Mix (Vazyme Biotech, Nanjing, China), 1 μL of forward primer RA2-F3 (10 μmol/L), 1 μL of reverse primer RA2-B3 (10 μmol/L), 1 μL of serially diluted pMD18T-RA plasmid DNA, and DNase-free ddH_2_O to a final volume of 25 μL.

### 2.9. Specificity Testing of the LAMP-LFD Assay

To evaluate the specificity of the LAMP-LFD assay for the detection of RA, a total of 13 common waterfowl pathogens were tested, including RA, *Escherichia coli* (*E. coli*), *Staphylococcus aureus* (*S. aureus*), *Salmonella* spp., Fowl cholera (FC), *Edwardsiella* spp., Duck plague virus (DPV), Duck circovirus (DuCV), Muscovy duck parvovirus (MDPV), Duck hepatitis virus (DHV), Avian influenza virus (AIV), Duck reovirus (DRV), and *Mycoplasma anatis* (*M. anatis*). DNase-free ddH_2_O was used as a negative control.

### 2.10. Repeatability Testing of the LAMP-LFD Assay

The repeatability of the LAMP-LFD assay was evaluated using the recombinant plasmid pMD18T-RA. Recombinant plasmid pMD18T-RA extracted at the same time was serially diluted to concentrations from 7.76 × 10^4^ to 7.76 × 10^1^ copies/μL, with DNase-free ddH_2_O included as a negative control. To assess intra-assay repeatability, each concentration was subjected to LAMP amplification under the optimized conditions, and the amplification products were analyzed by 2% agarose gel electrophoresis, phenol red-based visualization, and LFD detection. Each test was performed in triplicate. To evaluate inter-assay repeatability, the same four plasmid concentrations were tested in independent experiments conducted at 6-day intervals, and the products were analyzed using agarose gel electrophoresis, phenol red-based visualization, and LFD detection.

### 2.11. Detection of Clinical Samples

To assess the applicability and reliability of the developed method, a total of 30 clinical samples suspected of RA infection were collected. Genomic DNA was extracted from all samples and tested using the LAMP-LFD assay established in this study. Conventional PCR was performed in parallel as a reference method, and the consistency between the two methods was evaluated. For samples that tested positive by LAMP, bacterial isolation followed by sequencing analysis was further performed, confirming the presence of RA.

## 3. Results

### 3.1. Screening of LAMP-LFD Primer Sets

**Figure 2:**
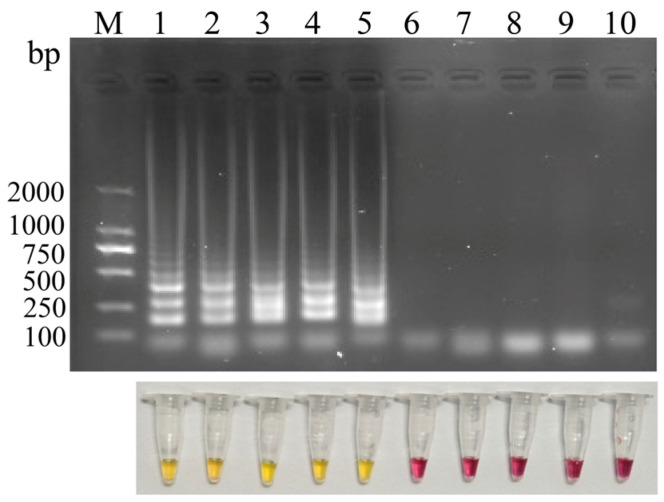
**Primer screening and evaluation of LAMP assay.** M, DL2000 DNA marker; lanes 1~5: primer sets 1, 2, 3, 4, and 5, respectively; lanes 6~10: negative controls for each primer sets, respectively.

Five candidate primer sets were screened by LAMP amplification, followed by analysis using agarose gel electrophoresis and phenol red-based colorimetric visualization. As shown in Figure 2, all five primer sets (Sets 1~5) generated typical ladder-like amplification patterns, while no amplification was observed in the negative controls. The phenol red-based colorimetric results were consistent with agarose gel electrophoresis analysis, further demonstrating that all primer sets exhibited acceptable amplification efficiency. Notably, primer set 2 produced the clearest and most prominent ladder-like banding pattern among all tested sets. Accordingly, primer set 2 was selected for subsequent analyses.

### 3.2. Optimized LAMP Reaction Conditions

**Figure 3:**
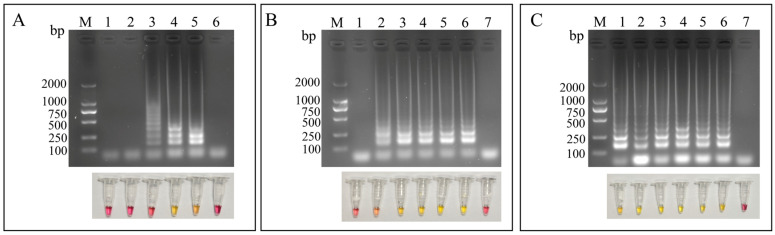
**Optimization of LAMP reaction conditions.** (**A**) Optimization of the ratio of inner and outer primers. M, DL2000 DNA marker; lanes 1~6: 1:1, 2:1, 4:1, 8:1, 16:1, and NTC. (**B**) Optimization of LAMP reaction time. M, DL2000 DNA marker; lanes 1~7: 10 min, 20 min, 30 min, 40 min, 50 min, 60 min, and NTC. (**C**) Optimization of LAMP reaction temperature. M, DL2000 DNA marker; lanes 1~7: 61 °C, 62 °C, 63 °C, 64 °C, 65 °C, 66 °C, and NTC.

The LAMP reaction conditions were optimized by evaluating the primer ratio, reaction time, and incubation temperature. As shown in Figure 3A, the clearest and most intense ladder-like amplification patterns were obtained when the ratio of inner to outer primers was 8:1. Ladder-like bands became detectable after 20 min of incubation, while the most distinct and intense bands were observed at 30 min (Figure 3B). Among the temperatures tested, 65 °C produced superior amplification performance, as indicated by clearer and brighter ladder-like bands (Figure 3C).

### 3.3. Sensitivity Analysis of the LAMP-LFD Assay

**Figure 4:**
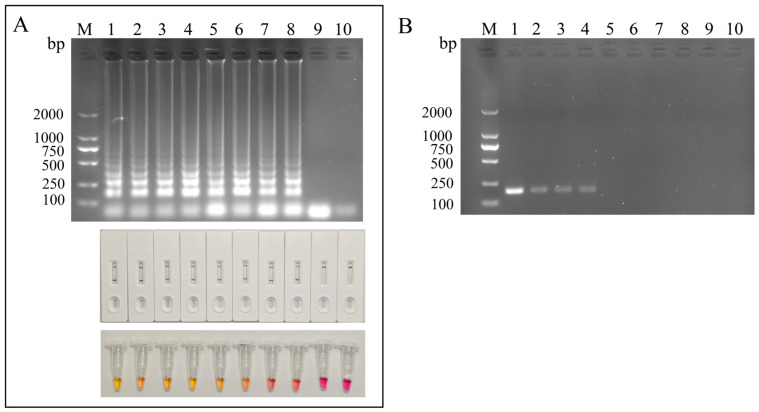
**Sensitivity of LAMP assay.** (**A**) Sensitivity of the LAMP assay. M, DL2000 DNA marker; lanes 1~10: 7.76 × 10^7^ copies/μL, 7.76 × 10^6^ copies/μL, 7.76 × 10^5^ copies/μL, 7.76 × 10^4^ copies/μL, 7.76 × 10^3^ copies/μL, 7.76 × 10^2^ copies/μL, 7.76 × 10^1^ copies/μL, 7.76 × 10^0^ copies/μL, 7.76 × 10^−1^ copies/μL, and NTC. (**B**) Sensitivity comparison of conventional PCR. M, DL2000 DNA marker; lanes 1~10: 7.76 × 10^5^ copies/μL, 7.76 × 10^4^ copies/μL, 7.76 × 10^3^ copies/μL, 7.76 × 10^2^ copies/μL, 7.76 × 10^1^ copies/μL, 7.76 × 10^0^ copies/μL, 7.76 × 10^−1^ copies/μL, 7.76 × 10^−2^ copies/μL, 7.76 × 10^−3^ copies/μL, and NTC.

Under the optimized LAMP reaction conditions, the sensitivity of the LAMP-LFD assay was evaluated with biotin-labeled FIP and 10-fold serial dilutions of pMD18T-RA plasmid DNA. Positive amplification signals were consistently detected by both LFD and agarose gel electrophoresis over a template concentration range from 7.76 × 10^7^ to 7.76 × 10^−2^ copies/μL, indicating a detection limit of 7.76 × 10^0^ copies/μL. By comparison, the detection limit of the LAMP assay coupled with phenol red-based colorimetric detection was 7.76 × 10^2^ copies/μL (Figure 4A). Conventional PCR amplification using primers RA2-F3 and RA2-B3 showed a detection limit of 7.76 × 10^2^ copies/μL (Figure 4B). These results indicate that the LAMP-LFD assay exhibits substantially higher sensitivity than conventional PCR.

### 3.4. Specificity of the LAMP-LFD Assay

**Figure 5:**
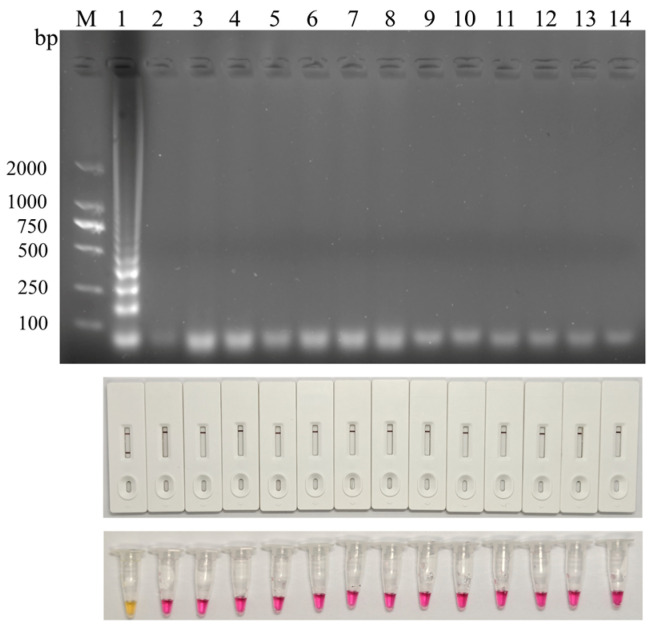
**Specificity of LAMP assay.** M, DL2000 DNA marker; lanes 1~14: RA, *Escherichia coli* (*E. coli*), *Staphylococcus aureus* (*S. aureus*), *Salmonella* spp., Fowl cholera (FC), *Edwardsiella* spp., Duck plague virus (DPV), Duck circovirus (DuCV), Muscovy duck parvovirus (MDPV), Duck hepatitis virus (DHV), Avian influenza virus (AIV), Duck reovirus (DRV), and *Mycoplasma anatis* (*M. anatis*), and NTC.

The specificity of the LAMP-LFD assay was evaluated using DNA from RA and a panel of common bacterial and viral pathogens, including *Escherichia coli* (*E. coli*), *Staphylococcus aureus* (*S. aureus*), *Salmonella* spp., Fowl cholera (FC), *Edwardsiella* spp., Duck plague virus (DPV), Duck circovirus (DuCV), Muscovy duck parvovirus (MDPV), Duck hepatitis virus (DHV), Avian influenza virus (AIV), Duck reovirus (DRV), and *Mycoplasma anatis* (*M. anatis*). Agarose gel electrophoresis revealed the characteristic ladder-like amplification bands only in the RA reaction, while no amplification products were detected for the other pathogens. Consistently, phenol red-based colorimetric detection showed an orange-yellow color change only in the RA reaction, whereas all other reactions remained magenta. After probe hybridization and LFD detection, a distinct test line was observed only in the RA sample, with no positive signal observed for the other pathogens (Figure 5). Collectively, these results indicate that the developed LAMP-LFD assay exhibits high specificity for the detection of RA.

### 3.5. Repeatability of the LAMP-LFD Assay

The repeatability of the established assay was evaluated by examining both intra-assay and inter-assay performance using four concentrations of pMD18T-RA. As shown in Table 2, all three detection methods exhibited 100% repeatability in both intra-assay and inter-assay evaluations, indicating that the LAMP-LFD assay developed in this study is highly repeatable and reliable.

### 3.6. Detection of Clinical Samples by LAMP-LFD

To further evaluate the clinical applicability of the established LAMP-LFD assay, a total of 30 clinical samples suspected of RA infection were examined using LAMP-LFD and conventional PCR. As summarized in Table 3, the positive detection rates obtained by conventional PCR, LAMP with agarose gel electrophoresis, LAMP-LFD, and LAMP with phenol red-based visual detection were 56.7% (17/30), 63.3% (19/30), 63.3% (19/30), and 60% (18/30), respectively. The detection rates of LAMP-LFD were identical to that of agarose gel electrophoresis-based LAMP analysis and slightly higher than that of phenol red-based visual detection. Overall, these results indicate that the LAMP-based visual detection method established in this study is suitable for the detection of clinical RA samples and exhibits higher sensitivity than conventional PCR.

## 4. Discussion

RA is an important bacterial pathogen in the waterfowl and poultry industry. In meat ducks, RA infection is typically characterized by pericarditis, perihepatitis, and airsacculitis [3]. Although RA has traditionally been regarded as a pathogen primarily affecting ducks, increasing reports of RA infection in chickens have been documented in recent years, indicating a potential expansion in host range. In chickens, RA infection has been associated with caseous obstruction of the oviduct and a marked reduction in egg production [4]. In addition, the extensive serotype diversity of RA, together with the lack of cross-protection among different serotypes, has contributed to its persistent and widespread dissemination [19,20,21]. Currently, various methods are available for RA detection, including bacterial isolation and culture, biochemical identification, ELISA, PCR, and qPCR [22,23,24,25]. Although these methods have demonstrated high sensitivity and specificity in laboratory settings, most molecular diagnostic approaches are labor-intensive, require expensive instrumentation, and depend on well-trained personnel and specialized laboratory facilities. Such constraints reduce their suitability for rapid field-based diagnosis, particularly in resource-limited settings. Therefore, the development of a simple, rapid, and reliable on-site detection method remains of considerable practical importance.

Previous studies have demonstrated that LAMP has been successfully applied to the detection of a wide range of animal pathogens, including highly pathogenic AIV [26], *Klebsiella pneumoniae* [27], porcine circovirus [28], foot-and-mouth disease virus [29], avian adenovirus [30], *Salmonella* spp. [31], and *Escherichia coli* [32]. Its successful application to the detection of bacterial, viral, and parasitic pathogens further underscores its robustness, versatility, and practical value in the field. Based on this solid foundation, the present study developed a LAMP-based assay for the rapid detection of RA. By targeting the highly conserved *ompA* gene [33], the assay provides broad detection coverage across different serotypes of RA. In an earlier study, *ompA* was used as the target gene for PCR-based detection of RA, yielding a specific 600-bp product from RA reference strains, while no amplification was observed in several non-target bacterial species, including *S. aureus*, *Streptococcus* spp., *Pasteurella multocida*, *Salmonella* spp., and *E. coli* [34]. Consistent with these findings, the phenol red- and LFD-based LAMP assay targeting *ompA* showed no cross-reactivity with other pathogens. Collectively, these results demonstrate that *ompA* is a highly specific and reliable molecular target for RA detection.

Furthermore, the visual LAMP-based detection method established in this study demonstrated higher sensitivity than previously reported diagnostic methods. Initially, Hou et al. [14] developed a colloidal gold immunochromatographic strip based on a monoclonal antibody against the RA GroEL protein, with a detection limit of 1 × 10^6^ CFU. Subsequently, Wu et al. [25] established a multiplex PCR assay for the rapid differentiation of serotype 1 and 2 strains of RA, achieving a detection limit of 10^2^ CFU. Zhang et al. [24] developed a qPCR assay targeting the *DtxR* gene, with a detection limit of 43 copies/μL. Notably, an RPA-LFD assay targeting *ompA* achieved a detection limit of 1.83 × 10^1^ copies/μL [35]. Similarly, Xie et al. [36] developed a qPCR assay targeting *ompA*, with a detection limit of 8 copies/μL. In contrast, the LFD-based LAMP assay developed in this study achieved a lower detection limit of 7.76 × 10^0^ copies/μL, indicating superior analytical sensitivity.

In the present study, the phenol red- and LFD-based LAMP assay demonstrated several advantages over existing diagnostic strategies. First, the phenol red- and LFD-based LAMP assay employs four to six specific primers targeting six distinct regions of the target gene. Amplification is initiated through the alternating action of inner and outer primers, generating dumbbell-shaped starting structures, followed by exponential amplification driven by strand displacement activity. This entire process can be completed under isothermal conditions at approximately 65 °C using only a simple water bath, without the need for sophisticated equipment. Second, although other isothermal amplification techniques, such as recombinase polymerase amplification (RPA), have shown certain advantages when using purified templates, their tolerance to inhibitors in crude samples remains controversial. Due to their complex enzyme systems and mild reaction conditions, RPA assays are particularly sensitive to inhibitors commonly present in crude samples, such as SDS and high concentrations of salts [37,38]. This limitation may be particularly problematic for tissue-derived specimens, such as duck tissues, which often require more rigorous nucleic acid purification prior to testing. Such additional processing not only reduces the practicality of on-site applications but may also increase the risk of false-negative results if purification is inadequate. In contrast, the LAMP assay established in this study operates at a relatively higher temperature (65 °C), which helps to mitigate the inhibitory effects of certain contaminants. Moreover, the *Bst* DNA polymerase used in LAMP has been widely validated for its robustness and tolerance to complex sample matrices. Therefore, while maintaining high sensitivity, this method likely requires less stringent sample pretreatment and is more suitable for rapid field-level diagnosis [39].

The phenol red- and LFD-based LAMP visual diagnostic methods are simple to perform and are readily applicable in field settings and resource-limited areas. A slight difference in the detection performance was observed during clinical sample testing, with the LAMP-LFD assay showing a positive rate approximately 3.3% higher than that of the LAMP-phenol red assay. This discrepancy is most likely attributable to the substantially higher analytical sensitivity of the LAMP-LFD assay, which was approximately 100-fold higher than that of the phenol red-based method. In addition, the phenol red-mediated color change may be less distinct in samples containing low target concentrations, thereby limiting endpoint interpretation near the detection threshold. In contrast, the LAMP-LFD assay incorporates an additional probe-based hybridization step prior to signal generation, which may enhance target capture and signal resolution, thereby providing superior analytical sensitivity [40].

Although the assay developed in this study demonstrated favorable sensitivity, specificity, and practical applicability for rapid detection of *RA*, several limitations should be acknowledged. First, while no cross-reactivity was observed with the microorganisms included in this study and in silico analysis further supported primer specificity, the current evaluation was based on a relatively limited panel of organisms and sequence datasets. Additional validation against a broader range of phylogenetically related bacteria and a more diverse collection of RA serotypes would further strengthen confidence in its species-level specificity and support its broader application in complex field settings. Second, although the assay showed stable performance under the tested conditions and good agreement with conventional PCR in clinical samples, the preliminary validation included only 30 clinical specimens, which limited the statistical power of the analysis and precluded a more rigorous assessment of diagnostic accuracy, agreement, and generalizability. Future studies incorporating larger and more diverse sample sets, ideally representing different sample types, infection stages, and geographic origins, will therefore be necessary to provide a more comprehensive evaluation of its clinical utility. In addition, as with other LAMP-based assays, the high yield of amplification products generated within a short time introduces an inherent risk of aerosol-mediated carryover contamination once reaction tubes are opened, which may result in false-positive findings if contamination control is insufficient [41]. Accordingly, strict procedural safeguards, including physical separation of pre- and post-amplification areas, dedicated equipment, aerosol-resistant filter tips, and appropriate handling and disposal of amplified products, are essential for preserving analytical reliability [42]. Taken together, although the present results support the promise of this assay as a rapid and field-deployable diagnostic tool, further large-scale validation and careful standardization of operating procedures will be important to fully establish its robustness and facilitate its routine application in laboratory and field settings.

## 5. Conclusions

In summary, a LAMP assay combined with phenol red-based colorimetric detection and LFD visualization was developed for the detection of RA by targeting the *ompA* gene. The entire detection process can be completed within 30 min, providing a time-efficient alternative to conventional molecular diagnostic methods. This method exhibits high specificity and sensitivity, with a detection limit of as low as 7.76 × 10^0^ copies/μL. Moreover, it demonstrated superior sensitivity and a higher detection rate in clinical samples compared with conventional PCR. Importantly, the assay overcomes key limitations of traditional detection methods, particularly the dependence on sophisticated instrumentation and skilled personnel. Results can be interpreted directly by visual observation of color changes in the reaction mixture or by visual assessment of the control/test lines (C/T lines) on the LFD following probe hybridization, thereby enabling convenient and user-friendly detection. Owing to its simplicity, rapidity, and high analytical performance, this method shows potential for point-of-care testing, field surveillance, and on-site diagnosis of RA.

## Figures and Tables

**Table 1:** The primers and probe of the LAMP assay for the detection of RA.

	Primer/Probe	Sequence (5′~3′)	Length (bp)
Set 1	RA1-F3	GTACTTTCTTAGTGGTAGGTCA	22
	RA1-B3	CATTCTCTCTTCGTTAGAAGC	21
	RA1-FIP	ACAGATGCAGCTCTTTCTCTAGATACGGATGTTAAGGGTAATGC	44
	RA1-BIP	AGCTAGAGGAGTTAATCCATCTCAAGACGCTGGTACTGTAGC	42
Set 2	RA2-F3	AGTGGTAGGTCATACGGAT	19
	RA2-B3	GTCCTTCATTCTCTCTTCGT	20
	RA2-FIP	Biotin-CAGCTACTACAGATGCAGCTCTTTTAAGGGTAATGCTAACTACAACT	47
	RA2-BIP	AGCTAGAGGAGTTAATCCATCTCATAGAAGCAGACGCTGGTA	42
Set 3	RA3-F3	CCAGTTGAAAACAACGGTTG	20
	RA3-B3	CATCGTACTGTGGAAGCC	18
	RA3-FIP	CCAGCTACATCAACACAAGCAGGCCAGATACAGATGGAGA	40
	RA3-BIP	CTGAAAACAATGGTTGTCCTTGGCAGGAACTGTAGGACACTT	42
Set 4	RA4-F3	GCATCTGTAGTAGCTGCTTTA	21
	RA4-B3	TCTTCACTACTGGAAGATCAG	21
	RA4-FIP	TGGTACTGTAGCTTCAGCAGAACGCTAGAGGAGTTAATCCATCTC	45
	RA4-BIP	CGTCTGCTTCTAACGAAGAGAGAATCTGAAGAGCTTCCCAAG	42
Set 5	RA5-F3	GCTTTAGAAGCTAGAGGAGTT	21
	RA5-B3	TCTTCACTACTGGAAGATCAG	21
	RA5-FIP	TCGTTAGAAGCAGACGCTGGATCTCAGTTAAAATCTAAAGGGGT	44
	RA5-BIP	AGAGAGAATGAAGGACAGAAAAGTGCTTCTGAAGAGCTTCCCAAG	45
Probe	RA2-HP	FAM-GTTGGTTCTGCTGAAGCTAC	20

**Table 2:** The repeatability of the LAMP assay.

Plasmid Concentration (Copies/μL)	Intra-Assay			Inter-Assay		
	No. of Tests	No. of Positives	Positive Rate	No. of Tests	No. of Positives	Positive Rate
7.76 × 10^4^	3	3	100%	3	3	100%
7.76 × 10^3^	3	3	100%	3	3	100%
7.76 × 10^2^	3	3	100%	3	3	100%
7.76 × 10^1^	3	3	100%	3	3	100%
NTC	3	0	0%	3	3	0%

**Table 3:** Comparison of the positive detection rates of LAMP-based assay and conventional PCR.

Methods	Detection Numbers	Positive Numbers	Positive Rate
LAMP-agarose gel electrophoresis	30	19	63.3% (19/30)
LAMP-LFD	30	19	63.3% (19/30)
LAMP-phenol red	30	18	60% (18/30)
Conventional PCR	30	17	56.7% (17/30)

## Data Availability

The original contributions presented in this study are included in the article. Further inquiries can be directed to the corresponding authors.
